# The landscape of neutralizing antibodies against SARS-CoV-2 variants: Insights from a stratified vaccination cohort

**DOI:** 10.3389/fimmu.2025.1667392

**Published:** 2025-09-26

**Authors:** Maria Julia Fialho, Carlos José Ferreira da Silva, Gabriel Brendo Alves Miranda, Izadora Sthephanie da Silva Assis, Adalgiza Mafra Moreno, Paulo Henrique Moura, Ana Carolina Proença da Fonseca, Tamara Silva, Sergian Vianna Cardozo

**Affiliations:** ^1^ Laboratory of Genetics, Postgraduate Program in Translational Biomedicine (BIOTRANS), University of Grande Rio (UNIGRANRIO), Rio de Janeiro, Brazil; ^2^ Laboratory of Technology in Biochemistry and Microbiology, State University of Rio de Janeiro, Rio de Janeiro, Brazil; ^3^ Human Genetics Laboratory, Oswaldo Cruz Foundation (IOC/FIOCRUZ), Rio de Janeiro, Brazil; ^4^ Laboratory of Immunopharmacology, Oswaldo Cruz Foundation (IOC/FIOCRUZ), Rio de Janeiro, Brazil; ^5^ Health and Aging Research Group, Iguaçu University (UNIG), Nova Iguaçu, Brazil; ^6^ Postgraduate Program in Physical Activity Sciences, Salgado de Oliveira University, Rio de Janeiro, Brazil; ^7^ Department of Genetics and Molecular Biology, Federal University of the State of Rio de Janeiro (UNIRIO), Rio de Janeiro, Brazil; ^8^ Laboratory of Cellular and Molecular Therapy and Physiology, State University of Rio de Janeiro (UERJ), Rio de Janeiro, Brazil

**Keywords:** COVID-19, vaccination, SARS-COV-2 variants, neutralizing antibodies, immune response

## Abstract

**Background:**

The COVID-19 pandemic has highlighted the crucial role of vaccines in preventing infection and reducing disease severity. However, emerging SARS-CoV-2 variants have posed challenges to vaccine-induced immunity.

**Objective:**

To evaluate the immunological response and clinical characteristics of individuals with complete and incomplete COVID-19 vaccination schedules, with a focus on neutralizing antibody levels against SARS-CoV-2 variants.

**Methods:**

A total of 245 participants were analyzed and stratified by complete and incomplete vaccination status. The complete vaccination was defined by 3 doses for <40 years or 4 doses for >40 years. Clinical data, serological responses, and neutralization levels against the Wuhan strain and the Delta and Omicron (BA.1, BA.2, BA.5) variants were evaluated.

**Results:**

Among the participants, 71 (29%) had an incomplete vaccination schedule, and 174 (71%) had completed the recommended doses. Despite only 118 (48.2%) of participants reporting a prior positive COVID-19 test, 210 (85.7%) tested positive for anti-nucleocapsid antibodies, underscoring a high rate of undiagnosed or asymptomatic infections. Neutralization levels were reduced in incompletely vaccinated individuals, especially against the Omicron BA.2 variant (89%). A moderate-to-strong correlation was found between declining immunity and increasing time since last vaccination or infection. Older participants demonstrated lower neutralization rates against the Wuhan and Delta strains, and cross-reactivity was observed between Wuhan and Delta (r ≈ 0.7), as well as between Omicron BA.1 and BA.2. Finally, a strong negative association was observed between time since the last known SARS-CoV-2 infection and neutralization against Omicron BA.2, as well as moderate-to-strong negative correlations with Omicron BA.5 and BA.1.

**Conclusion:**

COVID-19 vaccination is effective in eliciting protective immunity, although immune evasion by recent Omicron subvariants and waning immunity over time remain challenges. These findings support the need for updated booster strategies and continued public health efforts to ensure full vaccine coverage and long-term protection.

## 1 Introduction

SARS-CoV-2 is a single-stranded RNA virus belonging to the coronavirus group, capable of infecting humans through the respiratory system. Since the first recorded cases, it has been shown to be highly contagious and is responsible for causing the disease known as COVID-19. This disease can cause a distinct form of pneumonia that, in many cases, evolves into severe acute respiratory syndrome (SARS) (1).

In December 2019, a new strain of SARS-CoV emerged in the province of Wuhan, China, spreading rapidly, so much so that COVID-19 soon reached global proportions, leading the World Health Organization (WHO) to declare a pandemic in March 2020 (2). This disease has transformed global public health, impacting millions of lives. As of May 2025, over 777 million COVID-19 cases and more than 7 million deaths attributable to SARS-CoV-2 infection have been reported across 240 countries. The United States has reported the highest number of confirmed cases, followed by China and India (3). Although it is not among the countries with the highest number of reported cases, Brazil ranks second in the number of COVID-19-related deaths, with over 716.000 fatalities recorded (3, 4).

Since the emergence of the first reported cases, research on the novel coronavirus has advanced significantly (5).Viral infection initiates a host immune response characterized by the activation of macrophages and T lymphocytes - cellular immunity, which collectively function to constrain viral dissemination through the recognition and clearance of infected cells (6). The infection process may extend to other tissues and systems, such as the pulmonary system, triggering a pro-inflammatory cascade characterized by an exacerbated immune response, commonly referred to as cytokine release syndrome (7–9).

In some cases, the intensified immune response also contributes to a series of events that may result in thickening of alveolar walls, increased vascular permeability, and reduced pulmonary surfactant levels, ultimately leading to respiratory dysfunction - hallmark features of severe COVID-19 (10).

This tissue damage can manifest in several symptoms, such as fever, persistent cough, fatigue, muscle pain, and difficulty breathing, which can progress to pneumonia and severe acute respiratory syndrome, among others (11). Previous studies have demonstrated that the clinical outcome of COVID-19 can be negatively affected by pre-existing comorbidities such as cardiovascular diseases, obesity, diabetes, and immunosuppression, among others (12, 13).

Given the high mortality rate and rapid transmission of the virus, the pursuit of effective containment strategies and improvement of clinical outcomes has become a global scientific priority. The development and deployment of vaccines against SARS-CoV-2 were expedited, with the first COVID-19 vaccine introduced in July 2020. Thus, reducing infection rates, transmissibility, and the severity of clinical outcomes through vaccination became a fundamental step toward decreasing the morbidity, mortality, and spread of the disease (3, 14, 15).

Among the technologies used in vaccines that are still circulating in Brazil and approved by the WHO and ANVISA, some use synthetic mRNA as a platform (Comirnaty - Pfizer/Wyeth, Comirnaty bivalent BA.4/BA.5 - Pfizer, Spikevax bivalent and Spikevax - Adium/Moderna), viral vectors (Janssen Vaccine and COVID-19 Vaccine [recombinant] - Bio-Manguinhos/Fiocruz), and recombinant protein S + adjuvant (Zalika). However, although the aim of the vaccines against SARS-CoV-2 is the same, it is still unclear how durable the coverage generated by the vaccines will be (16). In this context, hybrid immunity, resulting from the combination of vaccination and prior SARS-CoV-2 infection, has been associated with a greater breadth and durability of neutralizing responses, in contrast to the suboptimal protection observed in individuals who were vaccinated alone (17).

As mentioned, several vaccines have been developed to combine agility, efficacy, and global applicability; however, targeting the immune response to reinfections by emerging variants must also be considered. In COVID-19, immunological imprinting - primary exposure to an antigen that shapes future immune responses - was evidenced by both reverse-boosting of antibodies against the seasonal coronaviruses OC43 and HKU1, and by the weaker response to Omicron BA.1 in individuals previously infected with Wuhan-Hu-1 (18).

Consistent with this, increased antibody rates against Wuhan-Hu-1 (ancestral) and a greater increase in antibodies against the XBB.1.5, EG.5.1, and JN.1 strains were observed in individuals immunized with the monovalent XBB.1.5 vaccine (19).

Although more than 13 billion doses of the vaccine have already been administered worldwide, part of the population remains reluctant to complete the vaccination schedule, as only 32% of the global population has been vaccinated with a booster dose (14). In Brazil, it is estimated that 86.65% of the population completed the primary vaccination schedule with up to two doses, while 19.71% received up to the fourth dose of the monovalent vaccine. Only 21.66% of the population was immunized with the fourth dose of the bivalent vaccine, showing low adherence and continuity in updating the vaccination schedule (20).

Notwithstanding the extensive implementation of vaccination programs, the immunological implication of incomplete vaccination regiments in the setting of co-circulating SARS-CoV-2 variants remain poorly defined, particularly among populations with high exposure risk. We postulate that individuals with incomplete vaccination display a compromised neutralizing capacity, most notably against emerging Omicron subvariants, and that the interval since the last immunological event (vaccination or infection) constitutes a critical determinant of this waning response.

This study aimed to quantify and compare neutralizing antibody responses against key SARS-CoV-2 variants in a cohort stratified by vaccination status (complete *vs*. incomplete) and to identify key clinical and temporal predictors of immune protection.

## 2 Materials and methods

### 2.1 Population and eligibility criteria

**Figure 1:**
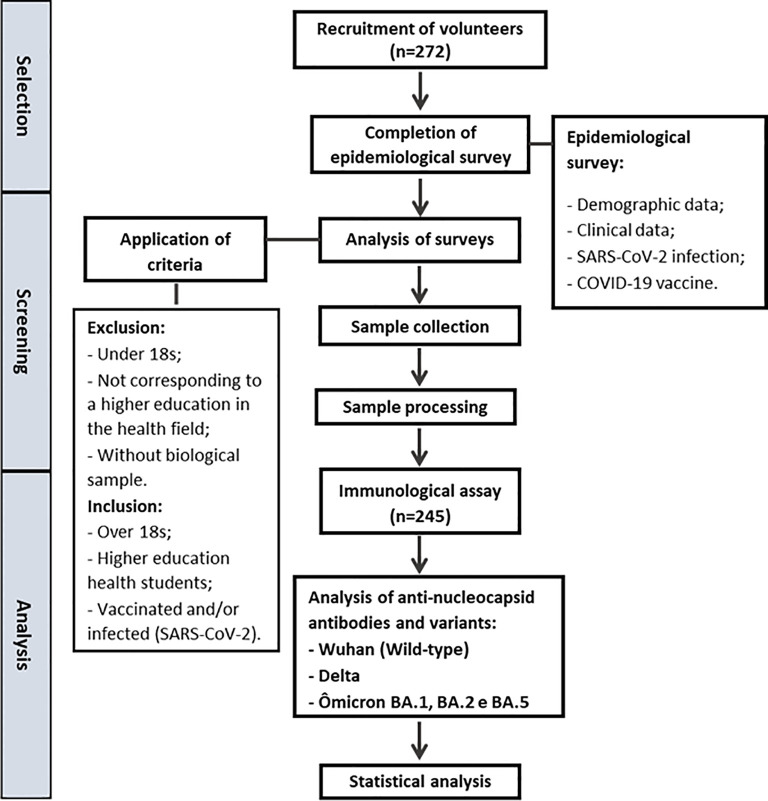
Study design. Flowchart representing the recruitment process and the methodologies used.

A cross-sectional study was carried out on a non-probabilistic sample of higher education students from Rio de Janeiro, Southeast Brazil (**Figure 1**). Health sciences students were selected due to their increased exposure to healthcare environments, which made them a relevant population for evaluating immune response patterns. A total of 272 volunteers were recruited between January and August 2024 through posters displayed on campus and by word of mouth. Participants were eligible if they had received one or more doses of the anti-SARS-CoV-2 vaccine or *had experienced* a positive COVID-19 episode. Briefly, the inclusion criteria were age (>18y) and performing a higher education in the health field. The biological samples that were not satisfactorily collected and/or processed were excluded from the study (n=27). The participants were stratified into two groups, complete (n=160) and incomplete (n=85) vaccination protocol. A complete vaccination protocol was considered as the administration of three or more doses of the vaccine for individuals up to 39 years of age, and four or more doses for individuals aged 40 years or older, according to the National Council of Health Secretaries in Brazil (21). All participants provided written informed consent prior to enrollment in the study. The study protocol was performed according to the Declaration of Helsinki (1964) and approved by the Ethics Committee of the University of Grande Rio – UNIGRANRIO/Afya (CAAE: 77130623.4.0000.5283).

### 2.2 Demographic and clinical variables

All information was self-reported and collected using *ad hoc* questionnaires developed by the research team (**Supplementary Material M1**). The questionnaires were applied by trained interviewers using standardized procedures. Age, sex, cigarette smoking status, body weight, height, use of medications, and history of clinical disease were obtained. Data of COVID-19 symptoms, vaccination, and treatment were collected from each participant.

Neutralizing responses were measured in participants with varying vaccination histories. For each participant, serum samples collected after the most recent vaccine dose were used to assess neutralization, without stratification by specific dose number.

### 2.3 Immunological analysis

Peripheral blood was collected from volunteers in tubes without anticoagulants to obtain serum for subsequent analyses (22). The microarray analysis was performed using the SARS-CoV-2 NT Chip^®^ Test Kit (V-NTCGOK - Viramed, Planegg, Germany) according to the manufacturer’s instructions. This immunoassay allows multiplex detection of anti-SARS-CoV-2 IgG antibodies and evaluates the neutralizing capacity of antibodies through the interaction between the RBD (Wuhan, Delta, and Omicron) and the human ACE2 receptor. Additionally, SARS-CoV-2 antigens N (nucleocapsid), RBD Wuhan (RBD-W), RBD Delta (RBD-D), and RBD Omicron (RBD-O) were also detected via binding antibodies in the same experiment. Each assay plate included both positive and negative controls: one negative control (kit standard), one internal negative control, and two internal positive controls with known values. Positivity thresholds were defined by the manufacturer as follows: RBD-1 (Wuhan), cutoff of 9%; RBDo-1 (original Omicron), cutoff of 29%; and N (nucleocapsid), cutoff of 74 arbitrary units (AU). For the Omicron subvariants (BA.2 and BA.5) and the Delta variant, no cutoff values were provided by the manufacturer. Therefore, any detectable neutralization was considered positive.

### 2.4 Statistical analysis

The normality of the variables was assessed using the Kolmogorov-Smirnov and Shapiro-Wilk tests. Quantitative and qualitative variables were described as median (interquartile range) and number (percentage), respectively. The quantity of IgG anti-S/RBD antibodies was considered the main outcome of interest and was evaluated both as a continuous and dichotomous variable (positive *vs*. negative). Additionally, qualitative detection of antibodies was considered.

To analyze the distribution of sociodemographic, clinical, and immunological data, the Mann-Whitney test was used for quantitative variables, and the chi-square test was used for categorical variables. Data were analyzed using SPSS Statistics software (version 23.0; IBM, New York, USA) with a significance level of 5%. Principal Component Analysis (PCA) and Spearman’s correlation plots were performed using the R programming language (v.4.3.2) and R-Studio software. A significance level of 0.05 was adopted.

## 3 Results

### 3.1 Clinical analysis

A total of 245 participants were included in this study after applying the inclusion and exclusion criteria. Participants were divided into two groups: those who had completed the COVID-19 vaccination schedule and those who had not completed it at the time of biological sample collection. Of the 245 participants, 160 (65.3%) had completed the vaccination cycle, and 85 (34.7%) had an incomplete cycle.

The sex distribution was 73.1% female and 26.9% male. Regarding clinical aspects, the most frequently reported conditions were respiratory diseases, obesity, anemia, and psychiatric disorders, with respiratory and psychiatric conditions showing the highest prevalence (**Supplementary Table S1**). Median age, weight, height, and smoking habits were very similar between the two groups (**Supplementary Table S2**).

When comparing the groups, individuals in the incomplete vaccination group most presented psychiatric conditions (12.9%), anemia (10.6%) and respiratory diseases (10.6%). In the complete vaccination group, the most frequent conditions were psychiatric disorders (16.9%), obesity (8.8%), and respiratory diseases (8.2%) (**Supplementary Table S1**).

Regarding vaccination status, only 17.6% of participants reported not having received any dose of the Pfizer vaccine, which is based on the messenger RNA (mRNA) platform. Pfizer was therefore considered the most widely administered vaccine, with some participants reporting up to five doses. Importantly, patients in the cohort had received a variable number of vaccine doses, ranging from one to six, which was taken into account in subsequent analyses. For the first dose, Pfizer was the most frequently used vaccine, followed by AstraZeneca and CoronaVac. This same order was maintained for the second and fourth doses; however, for the third dose, the Janssen vaccine was the third most administered. Among those who received a fifth dose, 91.9% reported having received Pfizer, followed by AstraZeneca (5.4%) and other vaccines (2.7%), including Moderna and Zalika. For the sixth dose, only the Pfizer vaccine was reported. Post-vaccination symptoms, possibly related to the vaccine, were reported by 44% of participants (**Table 1**).

**Table 1:** Vaccination aspects of the participants.

Variables	Total		Complete Vaccination		Incomplete vaccination		*p*
Vaccination schedule							
*Only the 1st dose*	245	10 (4.1%)	160	0 (0%)	85	10 (11.8%)	<0.001
*2 Doses*		67 (27.3%)		0 (0%)		67 (78.8%)	
*3 Doses*		8 (3.3%)		0 (0%)		8 (9.4%)	
*Complete Vaccination*		160 (65.3%		160 (100%)		0 (0%)	
1st dose of vaccine							
*Pfizer*	237	124 (52.3%)	154	76 (49.4%)	83	48 (57.8%)	0.356
*AstraZeneca*		55 (23.2%)		39 (25.3%)		16 (19.3%)	
*Janssen*		4 (1.7%)		2 (1.3%)		2 (2.4%)	
*CoronaVac*		53 (22.4%)		37 (24.0%)		16 (19.3%)	
*Other*		1 (0.4%)		0 (0%)		1 (1.2%)	
2nd dose of vaccine							
*Pfizer*	228	128 (56.1%)	154	81 (52.6%)	74	47 (63.5%)	0.297
*AstraZeneca*		48 (21.1%)		35 (22.7%)		13 (17.6%)	
*Janssen*		4 (1.8%)		3 (2%)		1 (1.4%)	
*CoronaVac*		47 (20.6%)		35 (22.7%)		12 (16.2%)	
*Other*		1 (0.4%)		0 (0%)		1 (1.3%)	
3rd dose of vaccine							
*Pfizer*	161	82 (50.9%)	153	80 (52.3%)	8	2 (25%)	0.127
*AstraZeneca*		47 (29.2%)		45 (29.4%)		2 (25%)	
*Janssen*		20 (12.4%)		17 (11.1%)		3 (37.5%)	
*CoronaVac*		12 (7.5%)		11 (7.2%)		1 (12.5%)	
4th dose of vaccine							
*Pfizer*	84	53 (63.1%)	84	53 (63.1%)	-	–	–
*AstraZeneca*		24 (28.6%)		24 (28.6%)		–	
*Janssen*		1 (1.2%)		1 (1.2%)		-	
*CoronaVac*		3 (3.6%)		3 (3.6%)		-	
*Other*		3 (3.5%)		3 (3.5%)		-	
5th dose of vaccine							
*Pfizer*	37	34 (91.9%)	37	34 (91.9%)	–	–	–
*AstraZeneca*		2 (5.4%)		2 (5.4%)		–	
*Other*		1 (2.7%)		1 (2.7%)		-	
6th dose of vaccine							
*Pfizer*	2	2 (100%)	2	2 (100%)	-	-	–
Number of Pfizer doses							
*0*	239	42 (17.6%)	156	14 (9.0%)	83	28 (33.7%)	<0.001
*1*		43 (18%)		30 (19.2%)		13 (15.7%)	
*2*		100 (41.8%)		58 (37.2%)		42 (50.6%)	
*3*		39 (16.3%)		39 (25.0%)		0 (0%)	
*4*		14 (5.9%)		14 (9.0%)		0 (0%)	
5		1 (0.4%)		1 (0.6%)		0 (0%)	
Number of AstraZeneca doses							
*0*	238	125 (52.5%)	155	61 (39.3%)	83	64 (77.1%)	<0.001
*1*		57 (23.9%)		50 (32.3%)		7 (8.4%)	
*2*		48 (20.2%)		36 (23.2%)		12 (14.5%)	
*3*		8 (3.4%)		8 (5.2%)		0 (0%)	
Number of Janssen doses							
*0*	240	214 (89.2%)	157	136 (86.6%)	83	78 (94%)	0.189
*1*		23 (9.6%)		19 (12.1%)		4 (4.8%)	
*2*		3 (1.2%)		2 (1.3%)		1 (1.2%)	
Number of CoronaVac doses							
*0*	238	176 (74.0%)	155	109 (70.3%)	83	67 (80.7%)	0.416
*1*		14 (5.9%)		10 (6.5%)		4 (4.8%)	
*2*		45 (18.9%)		34 (21.9%)		11 (13.3%)	
*3*		2 (0.8%)		1 (0.7%)		1 (1.2%)	
*4*		1 (0.4%)		1 (0.6%)		0 (0%)	
Number of doses of other types							
*0*	240	235 (97.9%)	157	153 (97.5%)	83	82 (98.8%)	0.134
*1*		4 (1.7%)		4 (2.5%)		0 (0%)	
*2*		1 (0.4%)		0 (0%)		1 (1.2%)	

(%) percentage; (<) less than; (p) probability of significance.

Stratification of the incomplete vaccination group by number of doses revealed that 10 (4.1%) participants had received only the first dose, 67 (27.3%) had received two doses, and 8 (3.3%) had received three doses (in cases where the recommended cycle was four doses).

Previous SARS-CoV-2 infection, confirmed by a positive COVID-19 test, was reported by 118 (48.2%) participants, with the majority (83%) reporting only one positive test. A statistically significant difference in the frequency of reported previous infection was observed between the two groups. As expected, most infections occurred in 2020 and 2021. While only 118 (48.2%) participants reported a prior positive COVID-19 test, anti-nucleocapsid antibodies were detected in 210 (85.7%) individuals, suggesting that nearly 40% of the cohort experienced undiagnosed or asymptomatic infection.

In total, 130 (53.1%) participants reported experiencing symptoms characteristic of COVID-19, including some who had no confirmed infection. Among them, 84 (64.6%) were from the complete vaccination group and 46 (35.4%) from the incomplete group.

Overall, mild symptoms were most frequently reported (58.9%); however, most individuals experienced three or more symptoms simultaneously. The most reported symptoms were loss of taste (60.3%), loss of smell (58.8%) and persistent cough (45.8%). Most participants reported home care as the primary intervention, although two individuals required hospitalization in a COVID-19 Unit, both belonged to the incomplete vaccination group. Among those who tested positive for COVID-19, 52 (21.2%) participants confirmed infection after receiving at least one vaccine dose, of which 38 (73.1%) were asymptomatic (**Supplementary Table S3**).

### 3.2 Immunological analysis

**Figure 2:**
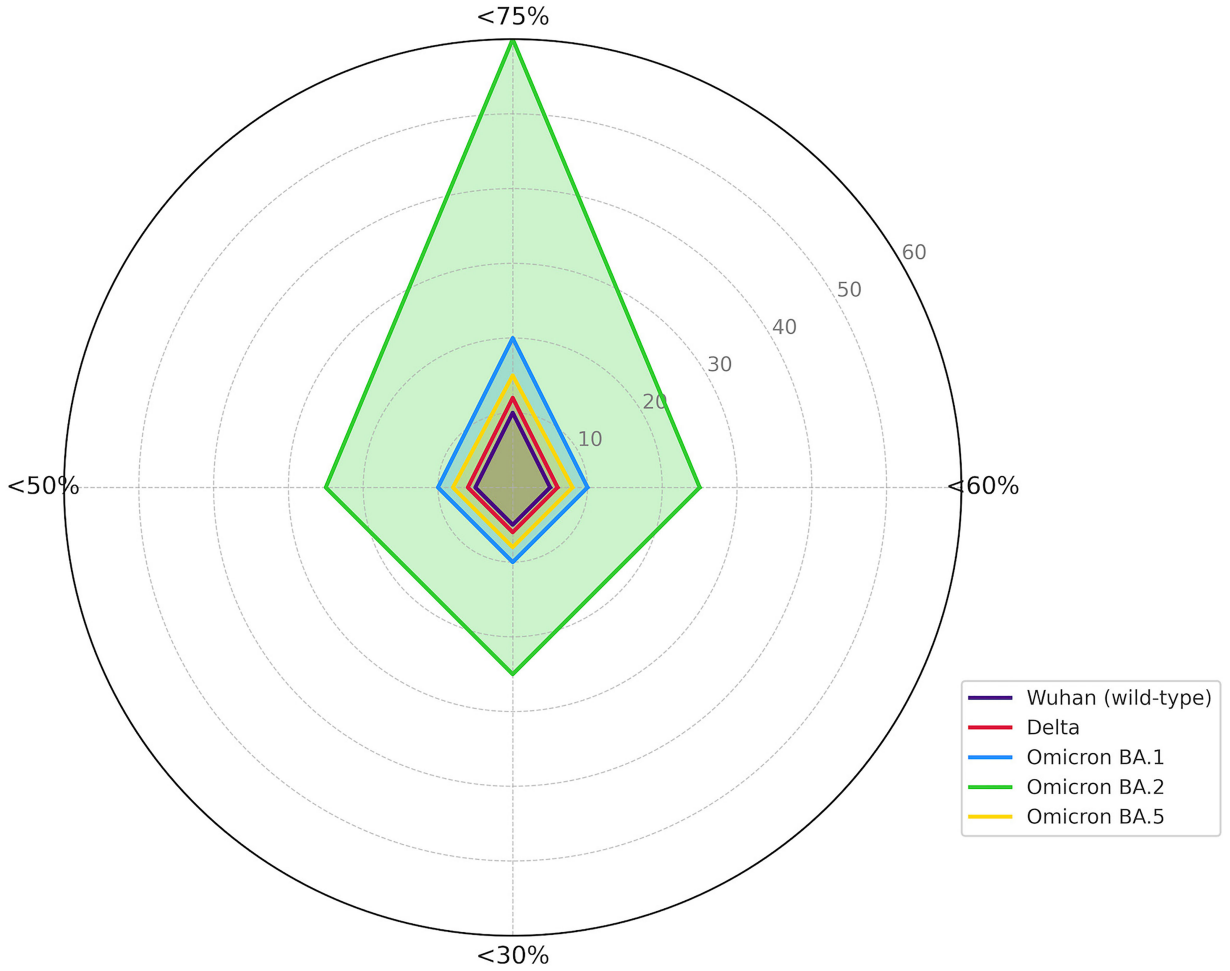
Reduction of the immune response by SARS-CoV-2 variant. (%) percentage; (<) less than.

Immunological analyses revealed that the median neutralization percentage and interquartile range for the Omicron BA.2 variant (70.5;100) were lower in participants with an incomplete vaccination schedule compared to the other strains tested, which showed 100% neutralization in both groups (100;100) (**Supplementary Table S4**, **Figure 2**). Furthermore, this variant had the highest number of individuals with reduced neutralization responses across all evaluated thresholds (**Figure 2**).

Neutralization rates above 75% were observed for the Delta, Omicron BA.5, Omicron BA.1 variants, and the original SARS-CoV-2 strain in most participants. However, Omicron BA.2 showed neutralization rates below 50% in five of the six individuals who also presented sub-threshold neutralization for Omicron BA.1. Similarly, four of the five participants with neutralization below 50% for Omicron BA.5 also showed reduced neutralization for Omicron BA.2 (**Supplementary Table S5**).

Although only 118 participants self-reported a previous positive test for COVID-19, serological testing identified 210 participants with anti-nucleocapsid antibodies. Notably, the Omicron BA.2 variant had the lowest neutralization rates among all variants tested, with 9.9% of participants exhibiting neutralization below 50% (**Supplementary Table S5**). Among these, 14 participants had completed the vaccination cycle, and 7 had received only two doses. Additionally, five participants who showed poor neutralization did not have detectable anti-nucleocapsid antibodies (**Supplementary Table S6**).

**Figure 3:**
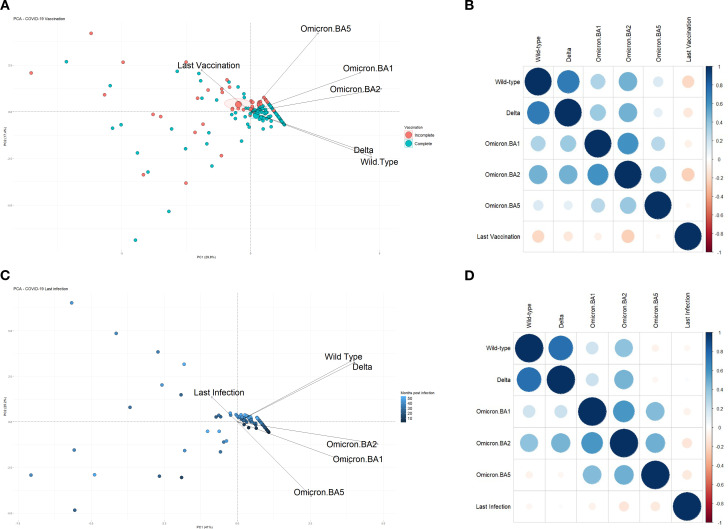
**(A)** Principal Component Analysis (PCA) plot showing the correlation between the number of months since the last administered vaccine dose and the neutralizing immune response against SARS-CoV-2 strains: Wuhan (wild-type), Delta, Omicron BA.1, BA.2, and BA.5. A total of 224 participants were included, with intervals ranging from 0 to 42 months since their last vaccine dose. **(B)** Correlation chart illustrating the relationship between the time since the last vaccination and the level of immunity observed for each of the analyzed variants. **(C)** PCA plot displaying the correlation between the number of months since the last reported SARS-CoV-2 infection and the resulting immunity against the same five variants. For this analysis, participants who did not report a previous infection were excluded, yielding a total of 115 individuals with infection intervals ranging from 2 to 54 months. **(D)** Correlation chart between the time since the last known infection and the immune response levels for each variant. Participants without complete data for the respective analyses were excluded from all graphs.

Multivariate analyses were conducted to evaluate the impact of various factors on the immunological outcomes. Principal Component Analysis (PCA) indicated that time since the last vaccine dose did not influence the neutralization capacity against the Omicron BA.5 variant (explained variance: 47.2%). However, this variable had a strong inverse relationship with neutralization of other SARS-CoV-2 strains, especially the Wuhan and Delta variants, suggesting a decline in antibody titers over time (**Figure 3A**).

This pattern was corroborated by correlation analysis (**Figure 3B**), which showed a strong positive correlation between neutralization of the Wuhan and Delta strains, indicating that both strains shared similar structures, which allows immune cells to neutralize them using a single strain as vaccine or a clonal base. These two variants also had moderate positive correlations with Omicron BA.2, and weaker correlations with Omicron BA.1 and BA.5, which denotes a higher degree of structural mutation of those strains, BA.1 and BA.5. Additionally, Omicron BA.1 and BA.2 demonstrated a strong mutual correlation, that either indicates mutual share of structures that facilitates a cross neutralization.

Correlation of neutralization capacity with time since the last vaccine dose (**Figure 3B**) also revealed a strong negative impact for the Wuhan and Omicron BA.2 strains, a moderate negative impact for Delta and Omicron BA.1, and a mild negative impact for Omicron BA.5.

Regarding time since the last known SARS-CoV-2 infection, PCA analysis (explained variance: 66.2%) showed a strong negative association with neutralization against Omicron BA.2, and moderate negative correlations with Omicron BA.5 and BA.1. However, neutralization of the Wuhan and Delta strains was unaffected by the interval since the last infection (**Figure 3C**).

**Figure 3D** further confirms these findings, revealing that neutralization of Omicron BA.2 and BA.5 was moderately impacted by time since last infection, while Wuhan and Omicron BA.1 showed mild negative correlations, and Delta exhibited no significant relationship. In this cohort, the Omicron BA.2 variant maintained a positive correlation with all other strains. The Wuhan and Delta strains also showed a strong positive correlation (Spearman’s p=0.7), and both exhibited moderate positive correlations with Omicron BA.2 and mild correlations with Omicron BA.1.

**Figure 4:**
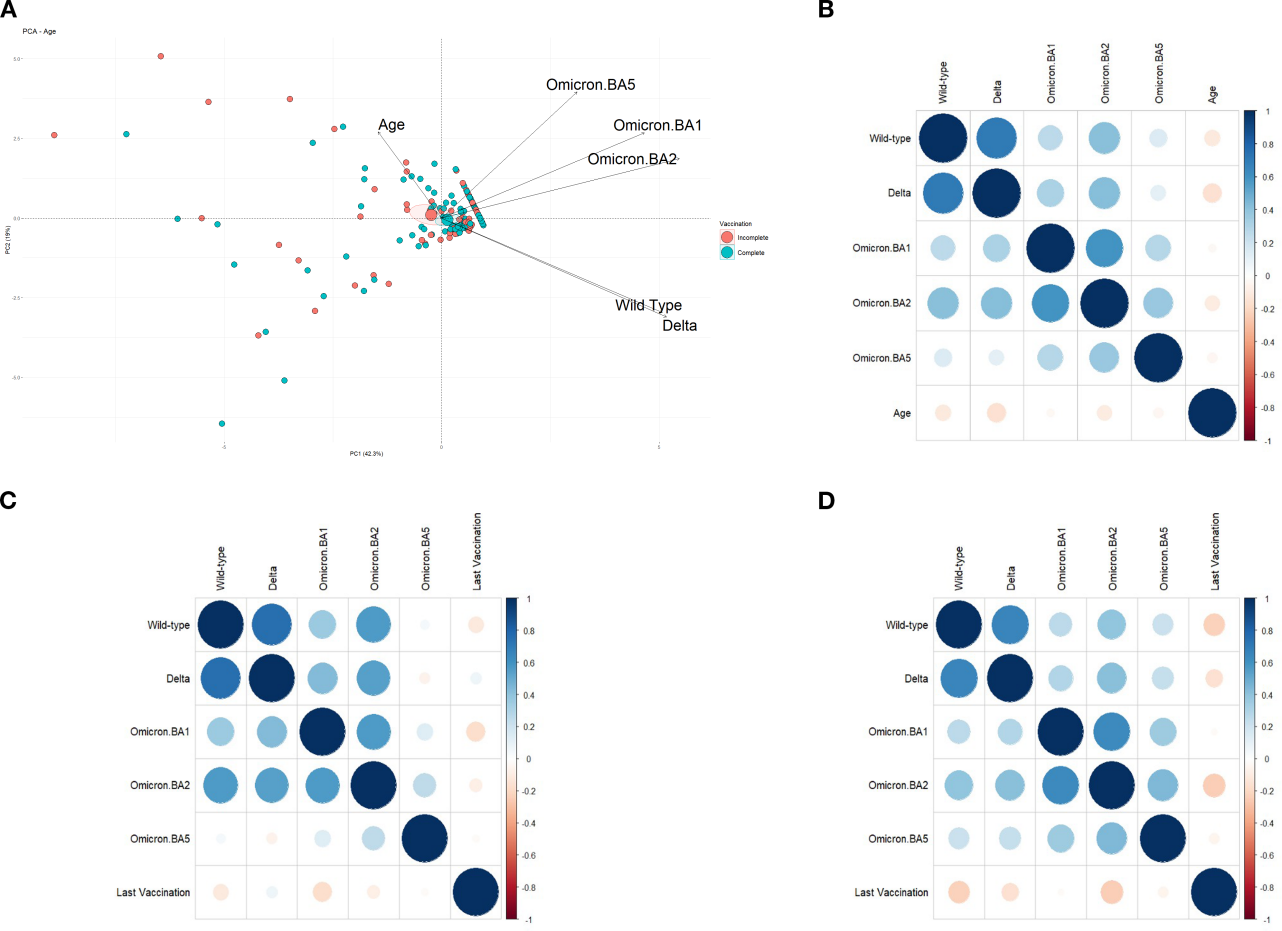
**(A)** Principal Component Analysis (PCA) plot showing the correlation between participant age and the neutralizing immune response against SARS-CoV-2 variants: Wuhan (wild-type), Delta, Omicron BA.1, BA.2, and BA.5. A total of 241 participants aged between 18 and 66 years were included in this analysis. **(B)** Correlation chart depicting the relationship between participant age and the level of immunity generated for each of the variants studied. **(C)** Correlation chart between the number of months since the last vaccine dose and the immune response observed in the incomplete vaccination subcohort. This analysis included 71 participants with an incomplete vaccination schedule. **(D)** Correlation chart between the number of months since the last vaccine dose and the immune response observed in the complete vaccination subcohort. This analysis included 153 participants with a complete vaccination schedule. Participants lacking complete data for the respective analyses were excluded from all graphs.

The impact of age on immunological responses was also evaluated. PCA (explained variance: 61.3%) indicated a strong negative correlation between age and neutralization of the Wuhan and Delta variants (**Figure 4A**). There was a mild negative correlation for Omicron BA.1 and BA.2, and no impact on the Omicron BA.5 variant. These findings suggest that increasing age was associated with reduced neutralizing responses to the Wuhan and Delta strains, and moderate decline for Omicron BA.1 and BA.2.

The correlation between age and neutralization for each strain is shown in **Figure 4B**. Negative correlations were observed for the Wuhan, Delta, and Omicron BA.2 variants, while Omicron BA.1 and BA.5 showed weaker negative correlations. Importantly, the strong positive correlations among variants were preserved across all subgroup analyses.

However, when analyzing the complete and incomplete vaccination groups separately, differences emerged. In the incomplete vaccination group, neutralization of the Omicron BA.5 variant had weak or even negative correlations with neutralization of the other strains, including a weak negative correlation with the Delta variant. Conversely, the complete vaccination group showed stronger inter-variant correlations.

Additionally, in the incomplete vaccination group, time since the last vaccine dose showed negative correlations with neutralization of the Wuhan and Omicron BA.1 strains, and a weak negative correlation with Omicron BA.2. In contrast, in the complete vaccination group, time since the last dose showed stronger negative correlations with Wuhan and BA.2, a negative correlation with Delta, and a mild negative correlation with BA.5 (**Figures 4C, D**).

## 4 Discussion

### 4.1 Summary of the main findings

The global public health importance of vaccination has been reaffirmed during the COVID-19 pandemic. International collaboration leading to the rapid development and deployment of vaccines represents a milestone in modern science. In this study, we confirmed that complete vaccination offers superior neutralizing activity compared to incomplete vaccination, particularly against Omicron subvariants. These results reinforce the effectiveness of vaccination in limiting SARS-CoV-2 transmission and disease progression, as reported in previous studies (23–25).

### 4.2 High rates of undiagnosed infection and hybrid immunity

One of the most striking findings was the considerable discrepancy between self-reported and serologically inferred infections. While 118 (48.2%) participants reported a prior positive COVID-19 test, anti-nucleocapsid antibodies were detected in 210 (85.7%), suggesting that nearly 40% of the cohort experienced undiagnosed or asymptomatic infection. This underreporting underscores the challenges of monitoring SARS-CoV-2 circulation. Moreover, the predominance of asymptomatic cases among vaccinated individuals who became infected reinforces the protective effect of vaccination. In this context, hybrid immunity, resulting from the combination of vaccination and prior infection, has been associated with broader and more durable neutralizing responses, highlighting the importance of considering both vaccination and infection history when evaluating immune protection.

A significant proportion of participants in our cohort reported a prior positive COVID-19 test (48.2%), but serological testing revealed anti-nucleocapsid antibodies in 85.7% of individuals. This discrepancy indicates that nearly 40% of infections were undiagnosed or asymptomatic, consistent with global reports highlighting the limitations of self-reported infection histories and routine testing strategies.

A striking finding in our cohort was the high prevalence of prior infection, which strongly suggests that hybrid immunity, derived from the combination of natural infection and vaccination, is the rule rather than the exception. This immune profile likely contributed to the magnitude and breadth of neutralizing responses observed, particularly in the fully vaccinated group, where repeated antigenic exposures may have enhanced cross-reactivity across variants. These observations are consistent with emerging evidence that hybrid immunity provides broader and more durable protection than vaccination or infection alone, and they underscore the importance of considering undocumented infection when evaluating vaccine effectiveness.

Together, these findings highlight that population-level immunity against SARS-CoV-2 cannot be accurately understood without accounting for undiagnosed infection. They also reinforce the need for continuous serological monitoring to capture the true extent of viral exposure and to better inform vaccine strategy and public health planning.

### 4.3 Immune escape by Omicron and waning immunity

Although vaccination conferred efficient protection against the ancestral Wuhan strain, reduced neutralization was observed against Gamma, Beta, Delta, and especially Omicron BA.1 and BA.2. These findings are consistent with other reports showing reduced vaccine-mediated neutralization against emerging variants (26–29). One possible explanation is the phenomenon of original antigenic sin, where immune memory preferentially targets the ancestral strain, limiting the generation of variant-specific antibodies. Neutralization capacity was also negatively influenced by the time elapsed since the last vaccine dose or infection, particularly for Omicron subvariants, supporting evidence of waning immunity over time (30–32). Importantly, these observations align with studies reporting vaccine effectiveness often falling below 90% against Omicron (33–36).

### 4.4 Novel and unexpected findings

Beyond confirming reduced neutralization against Omicron, our study revealed possible cross-protection patterns, such as between Wuhan and Delta strains, and between Omicron BA.1 and BA.2 subvariants. Additionally, a potential age-related decline in neutralizing titers was observed, consistent with prior evidence linking older age and comorbidities to reduced immune responses (37, 38). These findings highlight the multifactorial nature of immune protection, involving both host factors and viral evolution.

An unexpected and particularly relevant observation was the difference in cross-reactivity patterns between fully and incompletely vaccinated individuals. Complete vaccination appeared to promote a broader repertoire of neutralizing antibodies, likely due to repeated antigenic stimulation that enhances affinity maturation and expands recognition of conserved epitopes across variants. In contrast, incomplete vaccination may generate a narrower antibody response, with weaker inter-variant correlations and reduced cross-reactivity, especially against immune-evasive strains such as Omicron BA.2. Hybrid immunity could also contribute, as prior undiagnosed infection combined with vaccination may synergistically broaden antibody breadth. Furthermore, variations in epitope targeting (linear versus conformational) could help explain why certain participants displayed reduced neutralization against specific Omicron subvariants. Together, these mechanisms provide a biological basis for the differences observed and reinforce the importance of complete vaccination schedules to achieve robust and cross-variant protection.

### 4.5 Public health implications

The observed decline in neutralization, combined with immune escape of Omicron subvariants, highlights the importance of booster doses to sustain vaccine-induced immunity (39). Our findings also reveal that even within a cohort with privileged access to scientific information, vaccine hesitancy was present, as some participants resisted completing the vaccination schedule. This underscores the need for effective communication strategies to address vaccine hesitancy and to reinforce the public health value of complete vaccination (40–42).

### 4.6 Study limitations

This study has limitations that must be acknowledged. Differences in self-reported infection histories may have influenced antibody titers. We relied on a surrogate neutralization assay rather than the gold standard live virus assay, and did not assess cellular immunity, which plays a key role in protection against severe disease. The cross-sectional design also precluded longitudinal evaluation of immune responses. Finally, baseline differences between groups, such as the presence of cardiovascular comorbidities, may have influenced immune outcomes.

## 5 Conclusion

In conclusion, this study reinforces the importance and effectiveness of COVID-19 vaccination in generating a protective immune response, particularly in preventing symptomatic infection and reducing severe clinical outcomes. Despite this, a reduction in neutralization capacity against emerging SARS-CoV-2 variants especially Omicron BA.1 and BA.2 was observed, suggesting a potential for immune escape and the need for continued vigilance.

The negative correlation between neutralizing antibody levels and the time since the last vaccine dose or infection highlights the transience of the immune response, further emphasizing the importance of booster doses and adaptive immunization strategies. Additionally, our findings draw attention to the influence of age and comorbidities on the effectiveness of the humoral response, and to the persistence of vaccine hesitancy even among individuals with access to scientific information.

These results underscore the urgency of updating immunization policies to include variant-adapted vaccines and periodic monitoring of immune status. They also point to the need for continued public health education to increase vaccine acceptance and completion. Finally, longitudinal studies remain essential to better understand long-term immunity and to guide future vaccination strategies in the face of evolving viral dynamics.

## Data Availability

The raw data supporting the conclusions of this article will be made available by the authors, without undue reservation.
