# Supplementary material for: The landscape of neutralizing antibodies against SARS-CoV-2 variants: Insights from a stratified vaccination cohort
Source: Front Immunol. 2025 Sep 26;16:1667392. doi: 10.3389/fimmu.2025.1667392 (PMC12511102; doi:10.3389/fimmu.2025.1667392)

**Supplementary Material**

**Supplementary Material M1:** Epidemiological Survey of the study

**EPIDEMIOLOGICAL SURVEY**

Evaluation of genetic, inflammatory and anti-SARS-CoV-2 antibody biomarkers in health students vaccinated and/or infected with COVID-19.

There are no right or wrong answers; we just want to know your opinion on each item. Completing all items is crucial for the research team and the study's results.

Responses to the Survey are anonymous and confidential.

Participation is voluntary: you have the right to decide whether or not to participate and you can withdraw at any time, without any hassle.

Information about the study will be made available to participants who request it.

1. Participant ID number (to be filled in by the researcher responsible for collection) and Identity.
   1. **Participant’s name**
   2. **Telephone**
   3. **E-mail**
2. Do you agree to participate in the study?

( ) Yes

( ) No

1. What gender do you identify with?

( ) Feminine

( ) Masculine

( ) I'd rather not say

( ) Others

1. Identify your undergraduate course and period.
2. State your date of birth.
3. What is your nationality?
4. Indicate your approximate weight (kg).
5. Indicate your height (cm).
6. Do you smoke?

( ) Yes

( ) No

1. If you are a smoker, indicate how long (months, years).
2. If you are a smoker, indicate the number of cigarettes you smoke, on average, per day.

( ) 10 or less

( ) From 11 to 20

( ) From 21 to 30

( ) More than de 31

( ) Other. Specify:

1. If you have smoked in the past, indicate the number of years you smoked.
2. Indicate if you have any of these symptoms or diseases:

( ) Diabetes

( ) Obesity

( ) Asthma

( ) Heart failure

( ) Kidney failure

( ) High blood pressure

( ) Anemia

( ) Immunosuppression

( ) Sickle cell anemia

( ) Inflammatory diseases

( ) Oncological disease

( ) If you had cancer, how long ago did you finish treatment? ( ) Chronic obstructive pulmonary disease

( ) Other. Specify:

1. Do you use any medication?

( ) Yes

( ) No

1. If so, kindly indicate the medication used:

( ) Insulin

( ) Statins

( ) Antidepressants

( ) Immunosuppressants

( ) Antithrombotics/platelet aggregation

( ) Angiotensin-converting enzyme inhibitors (high blood pressure)

( ) Corticosteroids (also known as cortisone, anti-inflammatory action)

( ) Angiotensin II receptor blockers (Skin rash, cough, angioedema, hyperkalemia: patients with renal failure)

( ) Other. Specificy:

1. Have you had any surgical procedures or been hospitalized for any reason recently?

( ) Yes

( ) No

1. If so, please indicate the reason and date of hospitalization.
2. If you are a cancer patient, indicate how long you have been undergoing treatment. If you have completed treatment, indicate how long ago you completed that treatment.
3. Já Have you tested positive for COVID-19 (SARS-CoV-2)?

( ) Yes

( ) No

1. If so, please provide the month and year in which it occurred.
2. Did you have symptoms?

( ) Yes

( ) No

1. If so, have you had any of these symptoms?

( ) Fever (temperature of 38°C or higher) for more than 48 hours (the fever does not go down with medication, or the fever returns after a day without fever).

( ) Persistent cough (more than 5 days) or unusual cough.

( ) Difficulty breathing (shortness of breath at rest or with light exertion, breathing faster than usual, or purple mouth or fingernails).

( ) Recent chest or back pain (do not consider back pain already diagnosed or being treated).

( ) Persistent vomiting and/or severe diarrhea (three or more episodes in the same day, which interfere with your daily activities).

( ) Cough associated with headaches

( ) Cough associated with muscle pain

( ) Loss or change in taste

( ) Loss of smell

( ) Other. Specify: ________________________

1. If so, did you receive treatment?

( ) Admitted to the COVID Unit

( ) Admitted to the ICU

( ) Oxygen

( ) Mechanical ventilation

( ) Was intubated

( ) Other. Specify:

1. Have you been vaccinated?

( ) Yes

( ) No

( ) I do not intend to be vaccinated

1. If so, what is your vaccination schedule like?

( ) With the 1st dose

( ) Complete vaccination

( ) Was vaccinated with only 1 dose due to having had COVID-19

( ) Other. Specify:

1. If you have already been vaccinated, on what dates did you receive each dose (MONTH/YEAR)?
2. Did you have any symptoms after vaccination?

( ) Yes

( ) No

1. What brand of vaccine was administered to you? (Describe the order of doses on the side).

( ) Pfizer Vaccine

( ) AstraZeneca Oxford Vaccine

( ) Janssen (Janssen Biologics) Vaccine ( ) Coronavac Vaccine

( ) Other. Specify:

1. Have you been infected after being vaccinated?

( ) Yes

( ) No

1. If so, did you experience any symptoms? Please indicate which symptoms you experienced, taking into account those described above.
2. Have you been actively working in healthcare institutions during the pandemic?

( ) Yes

( ) No

1. If so, during what period?
2. What is your blood type and Rh factor?

( ) A

( ) B

( ) AB

( ) O

( ) Positive

( ) Negative

( ) I don’t know

1. Comments (to be completed by the researcher responsible for the collection).

**Supplementary Table S1.** Sociodemographic and clinical data of the study participants.

|  | **Total** | | **Complete Vaccination** | | **Incomplete Vaccination** | | **p** |
| --- | --- | --- | --- | --- | --- | --- | --- |
| **Gender** |  |  |  |  |  |  |  |
| *Male* | 245 | 66 (26.9%) | 160 | 47 (29.4%) | 85 | 19 (22.4%) | 0.238 |
| *Female* |  | 179 (73.1%) |  | 113 (70.6%) |  | 66 (77.6%) |  |
| **Age** | 242 | 23 (21;30) | 159 | 23 (21;29) | 83 | 23 (20;35) | 0.359 |
| **Smoking** |  |  |  |  |  |  |  |
| *No* | 245 | 237 (96.7%) | 160 | 154 (96.2%) | 85 | 83 (97.6%) | 0.558 |
| *Yes* |  | 8 (3.3%) |  | 6 (3.8%) |  | 2 (2.4%) |  |
| **Diabetes** |  |  |  |  |  |  |  |
| *No* | 245 | 234 (95.5%) | 160 | 154 (96.2%) | 85 | 80 (94.1%) | 0.443 |
| *Yes* |  | 11 (4.5%) |  | 6 (3.8%) |  | 5 (5.9%) |  |
| **Obesity** |  |  |  |  |  |  |  |
| *No* | 244 | 222 (91%) | 159 | 145 (91.2%) | 85 | 77 (90.6%) | 0.875 |
| *Yes* |  | 22 (9%) |  | 14 (8.8%) |  | 8 (9.4%) |  |
| **Heart Disease** |  |  |  |  |  |  |  |
| *No* | 244 | 232 (95.1%) | 159 | 155 (97.5%) | 85 | 77 (90.6%) | 0.018 |
| *Yes* |  | 12 (4.9%) |  | 4 (2.5%) |  | 8 (9.4%) |  |
| **Inflammatory Disease** |  |  |  |  |  |  |  |
| *No* | 244 | 235 (96.3%) | 159 | 154 (96.9%) | 85 | 81 (95.3%) | 0.538 |
| *Yes* |  | 9 (3.7%) |  | 5 (3.1%) |  | 4 (4.7%) |  |
| **Anemia** |  |  |  |  |  |  |  |
| *No* | 244 | 226 (92.6%) | 159 | 150 (94.3%) | 85 | 76 (89.4%) | 0.161 |
| *Yes* |  | 18 (7.4%) |  | 9 (5.7%) |  | 9 (10.6%) |  |
| **Immunosuppression** |  |  |  |  |  |  |  |
| *No* | 243 | 240 (98.8%) | 159 | 158 (99.4%) | 84 | 82 (97.6%) | 0.239 |
| *Yes* |  | 3 (1.2%) |  | 1 (0.6%) |  | 2 (2.4%) |  |
| **Autoimmune Disease** |  |  |  |  |  |  |  |
| *No* | 244 | 241 (98.8%) | 159 | 157 (98.7%) | 85 | 84 (98.8%) | 0.956 |
| *Yes* |  | 3 (1.2%) |  | 2 (1.3%) |  | 1 (1.2%) |  |
| **Respiratory Disease** |  |  |  |  |  |  |  |
| *No* | 244 | 222 (91%) | 159 | 146 (91.8%) | 85 | 76 (89.4%) | 0.531 |
| *Yes* |  | 22 (9%) |  | 13 (8.2%) |  | 9 (10.6%) |  |
| **Psychiatric Conditions** |  |  |  |  |  |  |  |
| *No* | 245 | 207 (84.5%) | 160 | 133 (83.1%) | 85 | 74 (87.1%) | 0.418 |
| *Yes* |  | 38 (15.5%) |  | 27 (16.9%) |  | 11 (12.9%) |  |
| **Other Conditions** |  |  |  |  |  |  |  |
| *No* | 243 | 220 (90.5%) | 159 | 145 (91.2%) | 84 | 75 (89.3%) | 0.629 |
| *Yes* |  | 23 (9.5%) |  | 14 (8.8%) |  | 9 (10.7%) |  |
| **Medication Use** |  |  |  |  |  |  |  |
| *No* | 244 | 152 (62.3%) | 159 | 97 (61.0%) | 85 | 55 (64.7%) | 0.570 |
| *Yes* |  | 92 (37.7%) |  | 62 (39.0%) |  | 30 (35.3%) |  |
| **Recent Surgery** |  |  |  |  |  |  |  |
| *No* | 245 | 227 (92.7%) | 160 | 146 (91.2%) | 85 | 81 (95.3%) | 0.248 |
| *Yes* |  | 18 (7.3%) |  | 14 (8.8%) |  | 4 (4.7%) |  |
| **Worked in a Healthcare Facility During the Pandemic** |  |  |  |  |  |  |  |
| *No* | 244 | 209 (85.7%) | 160 | 140 (87.5%) | 84 | 69 (82.1%) | 0.257 |
| *Yes* |  | 35 (14.3%) |  | 20 (12.5%) |  | 15 (17.9%) |  |
| **Blood Type** |  |  |  |  |  |  |  |
| *O+* | 205 | 74 (36.1%) | 133 | 47 (35.3%) | 72 | 27 (37.5%) | 0.773 |
| *O-* |  | 14 (6.9%) |  | 7 (5.3%) |  | 7 (9.7%) |  |
| *A+* |  | 81 (39.5%) |  | 55 (41.3%) |  | 26 (36.1%) |  |
| *A-* |  | 7 (3.4%) |  | 4 (3%) |  | 3 (4.2%) |  |
| *B+* |  | 16 (7.8%) |  | 11 (8.3%) |  | 5 (6.9%) |  |
| *B-* |  | 6 (2.9%) |  | 3 (2.3%) |  | 3 (4.2%) |  |
| *AB+* |  | 6 (2.9%) |  | 5 (3.8%) |  | 1 (1.4%) |  |
| *AB-* |  | 1 (0.5%) |  | 1 (0.7%) |  | 0 (0%) |  |

Legend: (%) percentage; (p) probability of significance; (+) positive Rh factor; (-) negative Rh factor.

**Supplementary Table S2.** Comparative data of median and interquartile range of characteristics between the groups.

|  | **Complete Vaccination** | | **Incomplete Vaccination** | |
| --- | --- | --- | --- | --- |
|  | **N** | **Median (Interquartile Interval)** | **N** | **Median (Interquartile Interval)** |
| **Age** | 159 | 23 (21;29) | 83 | 23 (20;35) |
| **Weight (kg)** | 159 | 69 (58;83) | 83 | 69 (60;83) |
| **Height (cm)** | 159 | 166 (160;173.5) | 83 | 165 (160;170) |
| If you smoke or have smoked: |  |  |  |  |
| **Months spent smoking** | 6 | 69 (12;150) | 3 | 36 (24) |
| **Number of cigarettes per day** | 6 | 10 (1.75;10) | 2 | 10 (10;10) |

Legend: (kg) kilograms; (cm) centimeters; (N) Demonstration number.

**Supplementary Table S3.** Participant-reported SARS-CoV-2 infection data.

|  | **Total** | | **Complete Vaccination** | | | **Incomplete vaccination** | | **p** |
| --- | --- | --- | --- | --- | --- | --- | --- | --- |
| **Tested positive for COVID-19** |  | | |  | |  | |  |
| *No* | 245 | 127 (51.8%) | 160 | | 84 (52.5%) | 85 | 43 (50.6%) | 0.776 |
| *Yes* |  | 118 (48.2%) |  |  | 76 (47.5%) |  | 42 (49.4%) |  |
| **2020** |  | | | | | | | |
| *No* | 245 | 199 (81.2%) | 160 | | 132 (82.5%) | 85 | 67 (78.8%) | 0.483 |
| *Yes* |  | 46 (18.8%) |  |  | 28 (17.5%) |  | 18 (21.2%) |  |
| **2021** |  | | | | | | | |
| *No* | 245 | 204 (83.3%) | 160 | | 131 (81.9%) | 85 | 73 (85.9%) | 0.424 |
| *Yes* |  | 41 (16.7%) |  |  | 29 (18.1%) |  | 12 (14.1%) |  |
| **2022** |  | | | | | | | |
| *No* | 245 | 205 (83.7%) | 160 | | 136 (85.0%) | 85 | 69 (81.2%) | 0.441 |
| *Yes* |  | 40 (16.3%) |  |  | 24 (15.0%) |  | 16 (18.8%) |  |
| **2023** |  | | | | | | | |
| *No* | 245 | 238 (97.1%) | 160 | | 156 (97.5%) | 85 | 82 (96.5%) | 0.645 |
| *Yes* |  | 7 (2.9%) |  |  | 4 (2.5%) |  | 3 (3.5%) |  |
| **2024** |  | | | | | | | |
| *No* | 245 | 241 (98.4%) | 160 | | 157 (98.1%) | 85 | 84 (98.8%) | 0.681 |
| *Yes* |  | 4 (1.6%) |  |  | 3 (1.9%) |  | 1 (1.2%) |  |
| **Times tested positive** |  | | | | | | | |
| *1* | 118 | 98 (83%) | 77 | | 62 (80.5%) | 41 | 36 (87.8%) | 0.02 |
| *2* |  | 16 (13.6%) |  |  | 14 (18.2%) |  | 2 (4.9%) |  |
| *3* |  | 3 (2.5%) |  |  | 0 (0%) |  | 3 (7.3%) |  |
| *4* |  | 1 (0.9%) |  |  | 1 (1.3%) |  | 0 (0%) |  |
| **Had symptoms** |  | | | | | | | |
| *No* | 245 | 115 (46.9%) | 160 | | 76 (47.5%) | 85 | 39 (45.9%) | 0.809 |
| *Yes* |  | 130 (53.1%) |  |  | 84 (52.5%) |  | 46 (54.1%) |  |
| **Symptom Severity** |  |  |  | |  |  |  |  |
| *Mild* | 129 | 76 (58.9%) | 83 | | 50 (60.3%) | 46 | 26 (56.5%) | 0.434 |
| *Moderate* |  | 9 (7.0%) |  |  | 4 (4.8%) |  | 5 (10.9%) |  |
| *Severe* |  | 44 (34.1%) |  |  | 29 (34.9%) |  | 15 (32.6%) |  |
| **Number of Symptoms** |  | | | | | | | |
| *1* | 129 | 15 (11.6%) | 83 | | 12 (14.5%) | 46 | 3 (6.5%) | 0.343 |
| *2* |  | 28 (21.7%) |  |  | 18 (21.7%) |  | 10 (21.7%) |  |
| *3* |  | 30 (23.3%) |  |  | 20 (24.1%) |  | 10 (21.7%) |  |
| *4* |  | 20 (15.5%) |  |  | 13 (15.7%) |  | 7 (15.2%) |  |
| *5* |  | 15 (11.6%) |  |  | 10 (12%) |  | 5 (10.9%) |  |
| *6* |  | 13 (10.1%) |  |  | 5 (6%) |  | 8 (17.4%) |  |
| *7* |  | 7 (5.4%) |  |  | 5 (6%) |  | 2 (4.4%) |  |
| *8* |  | 1 (0.8%) |  |  | 0 (0%) |  | 1 (2.2%) |  |
| **Treatment** |  |  |  | |  |  |  |  |
| *Home Care* | 54 | 50 (92.6%) | 37 | | 35 (94.6%) | 17 | 15 (88.2%) | 0.150 |
| *Admitted to the COVID-19 Unit* |  | 2 (3.7%) |  |  | 0 (0%) |  | 2 (11.8%) |  |
| *Oxygen* |  | 1 (1.8%) |  |  | 1 (2.7%) |  | 0 (0%) |  |
| *Other* |  | 1 (1.9%) |  |  | 1 (2.7%) |  | 0 (0%) |  |
| **Infected Post Vaccination** |  | | | | | | | |
| *No* | 245 | 193 (78.8%) | 160 | | 125 (78.1%) | 85 | 68 (80%) | 0.733 |
| *Yes* |  | 52 (21.2%) |  |  | 35 (21.9%) |  | 17 (20%) |  |
| **Symptom Severity** |  | | | | | | | |
| *Asymptomatic* | 52 | 38 (73.1%) | 35 | | 27 (77.1%) | 17 | 11 (64.7%) | 0.314 |
| *Mild* |  | 3 (5.8%) |  |  | 2 (5.7%) |  | 1 (5.9%) |  |
| *Moderate* |  | 4 (7.7%) |  |  | 1 (2.9%) |  | 3 (17.6%) |  |
| *Severe* |  | 7 (13.4%) |  |  | 5 (14.3%) |  | 2 (11.8%) |  |

Legend: (%) percentage; (p) probability of significance.

**Supplementary Table S4.**  Median and interquartile range data of immunological results between groups.

| **Strain** | **Complete Vaccination** | | **Incomplete vaccination** | |
| --- | --- | --- | --- | --- |
|  | **N** | **Median (interquartile interval)** | **N** | **Median (interquartile interval)** |
| Original (Wuhan) (%) | 160 | 100 (100;100) | 85 | 100 (100;100) |
| Delta (%) | 159 | 100 (100;100) | 85 | 100 (100;100) |
| Ômicron BA.1 (%) | 160 | 100 (100;100) | 85 | 100 (100;100) |
| Ômicron BA.2 (%) | 158 | 100 (78;100) | 85 | 89 (70.5;100) |
| Ômicron BA.5 (%) | 159 | 100 (100;100) | 85 | 100 (100;100) |
| Nucleocapsid (IU) | 147 | 298 (241;402) | 82 | 303 (200.75;412.50) |

Legend: (%) percentage; (N) sample number; (IU) International Units.

**Supplementary Table S5.** Comparative immunological data by percentage of neutralization and Arbitrary Units for nucleocapsid.

| **Strain** | **n total** | **<75%** | **<60%** | **<50%** | **< Cut-off** |
| --- | --- | --- | --- | --- | --- |
| **Wuhan (Original)** | 245 | 9 (3.7%) | 1(0.4%) | 0 | 0 |
| **Delta** | 244 | 5 (2%) | 0 | 0 | - |
| **Ômicron BA.1** | 245 | 18 (7.3%) | 18 (7.3%) | 15 (6.1%) | 6 (2.4%) |
| **Ômicron BA.2** | 243 | 58 (23.9%) | 33 (13.6%) | 24 (9.9%) | - |
| **Ômicron BA.5** | 244 | 9 (3.7%) | 9 (3.7%) | 5 (2%) | - |
| **Nucleocapsid** | 230 | - | - | - | 20 |

Legend: (n) sample number; (%) percentage.

**Supplementary Table S6.** Anti-nucleocapsid and vaccine immune profile of participants with neutralization rate below 50% for Omicron BA.2 variant.

| **Participants**  **(n = 24)** | **Vaccination cycle** | **Total doses of immunizer** | **Types of immunizers received** | **anti-nucleocapsid (AU)** |
| --- | --- | --- | --- | --- |
| 17 | Incomplete | 2 doses | Pfizer | Positive (292) |
| 27 | Complete | 5 doses | Coronavac, Pfizer e Astrazeneca | Positive (341) |
| 67 | Incomplete | 2 doses | Coronavac | Positive (229) |
| 69 | Incomplete | 2 doses | Pfizer | Positive (89) |
| 70 | Complete | 4 doses | Pfizer e Coronavac | Positive (241) |
| 71 | Incomplete | 1 dose | Pfizer | Negative (0) |
| 93 | Incomplete | 3 doses | Coronavac e Janssen | Positive (337) |
| 115 | Incomplete | 2 doses | Pfizer | Positive (305) |
| 127 | Incomplete | 1 dose | Pfizer | Negative (0) |
| 139 | Complete | 3 doses | Pfizer e Coronavac | Positive (432) |
| 145 | Complete | 4 doses | Pfizer e Astrazeneca | Positive (327) |
| 151 | Complete | 5 doses | Coronavac, Pfizer e Astrazeneca | Positive (230) |
| 180 | Complete | 4 doses | Pfizer | Positive (279) |
| 202 | Complete | 3 doses | Pfizer e Coronavac | Positive (570) |
| 217 | Complete | 3 doses | Pfizer e Coronavac | Positive (454) |
| 220 | Complete | 3 doses | Pfizer e Astrazeneca | Positive (82) |
| 227 | Complete | 3 doses | Pfizer e Astrazeneca | Negative (46) |
| 233 | Incomplete | 2 doses | Coronavac | Positive (243) |
| 234 | Incomplete | 2 doses | Pfizer | Positive (203) |
| 249 | Complete | 4 doses | Pfizer e Astrazeneca | Positive (271) |
| 256 | Complete | 3 doses | Pfizer e Astrazeneca | Negative (50) |
| 258 | Incomplete | 2 doses | Coronavac | Positive (305) |
| 264 | Complete | 5 doses | Pfizer e Astrazeneca | Negative (18) |
| 270 | Complete | 5 doses | Pfizer e Coronavac | Positive (257) |

Legend: (n) total number of the sample and (AU) Arbitrary Units.

**Supplementary Graph S1:** Neutralization rate according to the degree of vaccination


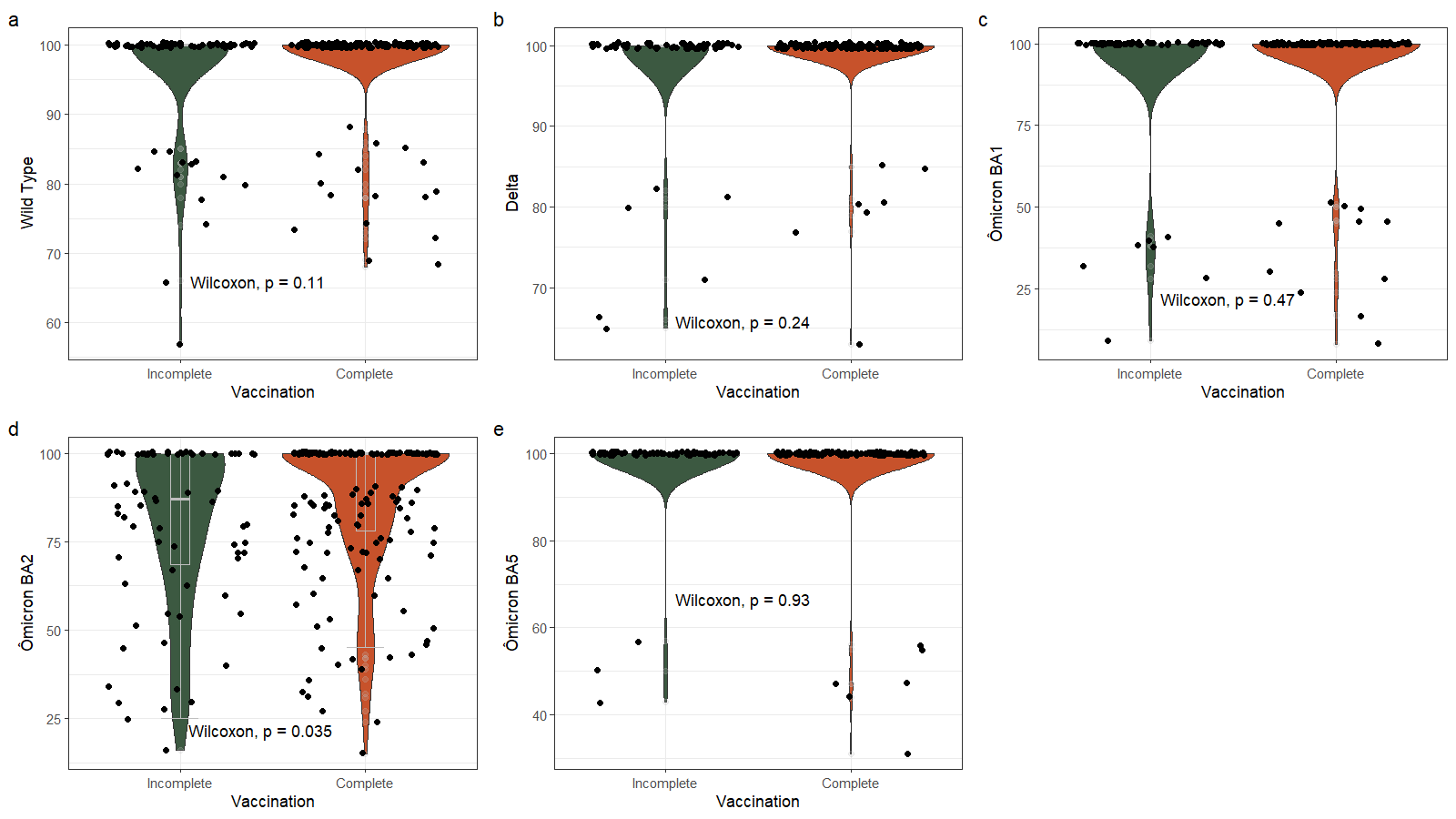

Supplement: Supplementary file 1 [file Table1.docx]
